# Adjuvant tyrosine kinase inhibitors plus anti-PD-1 therapy reduce recurrence in high-risk hepatocellular carcinoma after resection

**DOI:** 10.3389/fonc.2025.1710602

**Published:** 2025-11-13

**Authors:** Jiaxin Li, Yukun Sun, Ziniu Liu, Jianwen Huang, Jian Wu, Yun Huang, Yali Gao, Baomin Chen, Qiang He

**Affiliations:** 1 Department of Radiation Oncology, The First Affiliated Hospital, Sun Yat-sen University, Guangzhou, China; 2 Center of Hepato-Pancreato-Biliary Surgery, The First Affiliated Hospital, Sun Yat-sen University, Guangzhou, China; 3 Department of Dermatology, The First Affiliated Hospital, Sun Yat-sen University, Guangzhou, China

**Keywords:** hepatocellular carcinoma, high risk of recurrence, tyrosine kinase inhibitors (TKIs), anti-PD-1 antibodies, postoperative adjuvant therapy

## Abstract

**Background/aims:**

No standardized adjuvant treatment has been established for patients with hepatocellular carcinoma (HCC) following curative resection. This study aimed to evaluate the efficacy and safety of adjuvant tyrosine kinase inhibitors (TKIs) in combination with anti-programmed death-1 (PD-1) antibody in HCC patients with high-risk factors for recurrence.

**Methods:**

HCC patients who underwent hepatectomy at the First Affiliated Hospital, Sun Yat-sen University between January 2020 and December 2022 were retrospectively enrolled. Baseline differences were balanced between HCC patients with adjuvant therapy group (TKIs+PD-1, AT group) and hepatectomy alone group (HA group) by propensity-score matching (PSM). Recurrence-free survival (RFS) and overall survival (OS) were compared between these two groups. Univariable and multivariable analyses were used to identify prognostic factors, and a subgroup analysis was also conducted to assess treatment efficacy across different patient subpopulations.

**Results:**

A total of 357 HCC patients with high risk of recurrence was enrolled. After PSM, 50 matched pairs of patients were analyzed. After PSM, the median follow-up was 30.2 months (IQR 18.47–37.48). The median RFS was 25.77 months (95% CI: 15.07- not evaluated (NE)) in the AT group versus 7.7 months (95% CI: 5.43-15.2) in the HA group. The AT group demonstrated significantly longer RFS compared with the HA group (*P* = 0.0029), while no significant difference in OS was observed (*P* = 0.62). Multivariable analyses identified adjuvant therapy with TKIs and anti-PD-1 antibodies as an independent protective factor for RFS, but not for OS. Subgroup analysis further confirmed the RFS benefit of adjuvant combination therapy in patients with high-risk factors, without a corresponding improvement in OS.

**Conclusions:**

Adjuvant TKIs combined with anti-PD-1 antibody significantly prolongs recurrence-free survival in HCC patients with high risk of postoperative recurrence. However, this combination does not confer a survival benefit in terms of overall survival. These findings support the potential clinical utility of adjuvant targeted immunotherapy in this high-risk population and highlight the need for further validation in prospective, randomized studies.

## Introduction

Hepatocellular carcinoma (HCC) accounts for approximately 75%-85% of primary liver cancers and ranks as the third leading cause of cancer-related death globally (1). Hepatectomy remains the primary curative treatment for early-stage HCC. However, despite curative intent, postoperative recurrence remains a major challenge, with up to 70% of patients experiencing tumor recurrence within five years, and the 5-year overall survival rate remains below 50% (2, 3). Prognosis is particularly poor for patients with high-risk pathological and clinical features, such as microvascular invasion (MVI), satellite nodules, multifocal tumors, hepatic vein tumor thrombus (HVTT), portal vein tumor thrombus (PVTT), positive surgical margins, elevated alpha-fetoprotein (AFP ≥ 400 ng/mL), and large tumor size (particularly >5 cm) (4–8). Therefore, there is a critical need for effective postoperative adjuvant therapies to reduce recurrence risk and improve long-term outcomes in this high-risk population.

Currently, no universally accepted adjuvant therapy has been established for HCC following resection. Nevertheless, recent advances in targeted therapy and immunotherapy have opened new avenues for improving postoperative outcomes (9). Sorafenib, a tyrosine kinase inhibitor, has shown potential in reducing recurrence in several retrospective studies (10–13). However, a phase III randomized controlled trial evaluating sorafenib as adjuvant therapy following surgical resection or local ablation failed to meet its primary endpoint of recurrence-free survival (RFS) improvement (14).

Immune checkpoint inhibitors (ICIs), including antibodies targeting programmed death 1 (PD-1) and its ligand (PD-L1), have demonstrated the ability to restore anti-tumor immunity by enhancing T cell-mediated tumor recognition (15). In a randomized phase II trial, sintilimab (an anti-PD-1 antibody) significantly prolonged RFS compared to active surveillance in patients with high-risk features following curative resection (16). Additional studies have also reported improved survival outcomes with adjuvant anti-PD-1 therapy in high-risk HCC cohorts (17, 18). Furthermore, the IMbrave050 phase III trial provided the efficacy of targeted combined with immunotherapy as adjuvant therapy for HCC (19). Although interim results of IMbrave050 supported adjuvant atezolizumab plus bevacizumab and led to guideline inclusion, the final analysis showed a diminished RFS benefit and no OS improvement.

Herein, we conducted a retrospective real-world study to evaluate the efficacy and safety of adjuvant TKIs combined with anti-PD-1 antibodies, compared with hepatectomy alone, in HCC patients at high risk of postoperative recurrence.

## Materials and methods

### Patient selection

The study obtained full ethical approval from The Ethics Committee of the First Affiliated Hospital of Sun Yat-sen University. Patient consent was waived due to the retrospective nature of the study. This retrospective study collected data from HCC patients who underwent radical resection at the First Affiliated Hospital, Sun Yat-sen University between January 2020 and December 2022.

The inclusion criteria were as follows: 1) age ≥ 18 years; 2) histopathology confirmed HCC; 3) HCC patients underwent R0 resection; 4) Child-Pugh A or B; 5) No prior systemic anti-tumor treatment before radical hepatectomy; 6) no residual or recurrent tumor on postoperative radiology at 4–6 weeks after hepatectomy; 7) at least one high risk factor for recurrence: MVI, PVTT, HVTT, satellite nodules, tumor nodules > 3, AFP > 400 ng/ml, or maximum tumor size > 5 cm. The exclusion criteria were as follows: 1) sarcomatoid HCC, HCC mixed with cholangiocarcinoma, or fibrolamellar HCC; 2) history of other malignancies; 3) incomplete follow-up records; (4) non-compliance with adjuvant therapy.

### Definition of high risk of recurrence

MVI (Microvascular Invasion) is defined as clusters of cancer cells within the lumens of vessels lined with endothelial cells observed under a microscope (20). Satellite nodules are small tumor foci within the liver tissue adjacent to the primary tumor, with a distance of less than 2 centimeters from the primary tumor (21). PVTT (Portal Vein Tumor Thrombosis) and HVTT (Hepatic Vein Tumor Thrombus) involve tumor emboli within the portal vein and hepatic vein, respectively.

### Adjuvant TKIs combined with anti-PD-1 antibody treatment

HCC patients with at least one high recurrence risk factor are advised to receive adjuvant therapy after liver resection. However, the final treatment decision depends on the patient and their family. For patients receiving adjuvant therapy, the TKIs included sorafenib, lenvatinib, apatinib, and anlotinib. The anti-PD-1 antibodies used were camrelizumab, tislelizumab, pembrolizumab, and sintilimab. TKIs and anti-PD-1 antibodies were administered at recommended dosages from four weeks after surgery for up to one year, or until HCC recurrence or unacceptable toxicity, whichever occurred first. Intermittent or reduced dosage were allowed during treatment to decrease drug-related toxicities. Adverse events were classified according to the National Cancer Institute Common Terminology Criteria for Adverse Events version 5.0.

The study divided all patients into two groups based on adjuvant therapy acceptance: (1) HCC patients who received TKIs combined with anti-PD-1 antibody therapy after hepatectomy (adjuvant therapy group, AT group); and (2) HCC patients who underwent hepatectomy alone without adjuvant therapy (hepatectomy alone group, HA group).

### Postoperative follow-up and clinical outcomes

HCC patients in both groups received regular follow-ups after liver surgery. The first follow-up occurred 4–6 weeks after hepatectomy, then every 2–3 months for two years. Follow-up was then performed every 6 months. Follow-up examinations included physical examination, laboratory tests (peripheral blood test, liver function, AFP), and abdominal radiological examinations (ultrasound, contrast enhanced CT, or MRI). Recurrence was diagnosed based on typical HCC imaging findings, with or without persistently elevated serum AFP-levels. RFS was defined as the time from hepatectomy to tumor recurrence or last follow-up. Overall survival (OS) was defined as the time from surgery to death or last follow-up. The last follow-up date for this study was December 31, 2023.

### Statistical analysis

Statistical analysis was performed using R software version 4.3.2 (http://www.R-project.org). Continuous variables with a normal distribution were presented as mean ± standard deviation (SD), while those without were reported as medians and interquartile ranges (IQR). Independent samples t-tests or Mann-Whitney U tests were used to compare differences between continuous variables, depending on the specific circumstances. Categorical variables were presented as numbers (n) or percentages (%), and compared using chi-squared test or Fisher’s exact test. To adjust for confounding factors, a 1:1 propensity score matching (PSM) was performed. Propensity scores, ranging from 0 to 1, were generated using binary logistic regression with selected variables. Nearest-neighbor matching was used to match patients in the AT group to those in the HA group. Pairs were matched within a propensity-score logit range of 0.16 SD. A standardized mean difference (SMD) below 0.1 indicates a meaningful balance in baseline covariate (22, 23). Survival curves were estimated using the Kaplan-Meier method and compared with the Log rank test. Univariate and multivariate Cox regression analyses determined independent prognostic factors for RFS and OS. In the univariate Cox regression, only factors with a *p*-value less than 0.05 were included in the multivariate analysis. Subgroup survival analysis was conducted using univariate Cox regression, stratified by clinical variables, and forest plots were drawn with hazard ratio (HR) and 95% confidence interval (CI). The differences between groups with a two-tailed *p* < 0.05 were considered statistically significant.

## Results

### Patient characteristics

**Figure 1:**
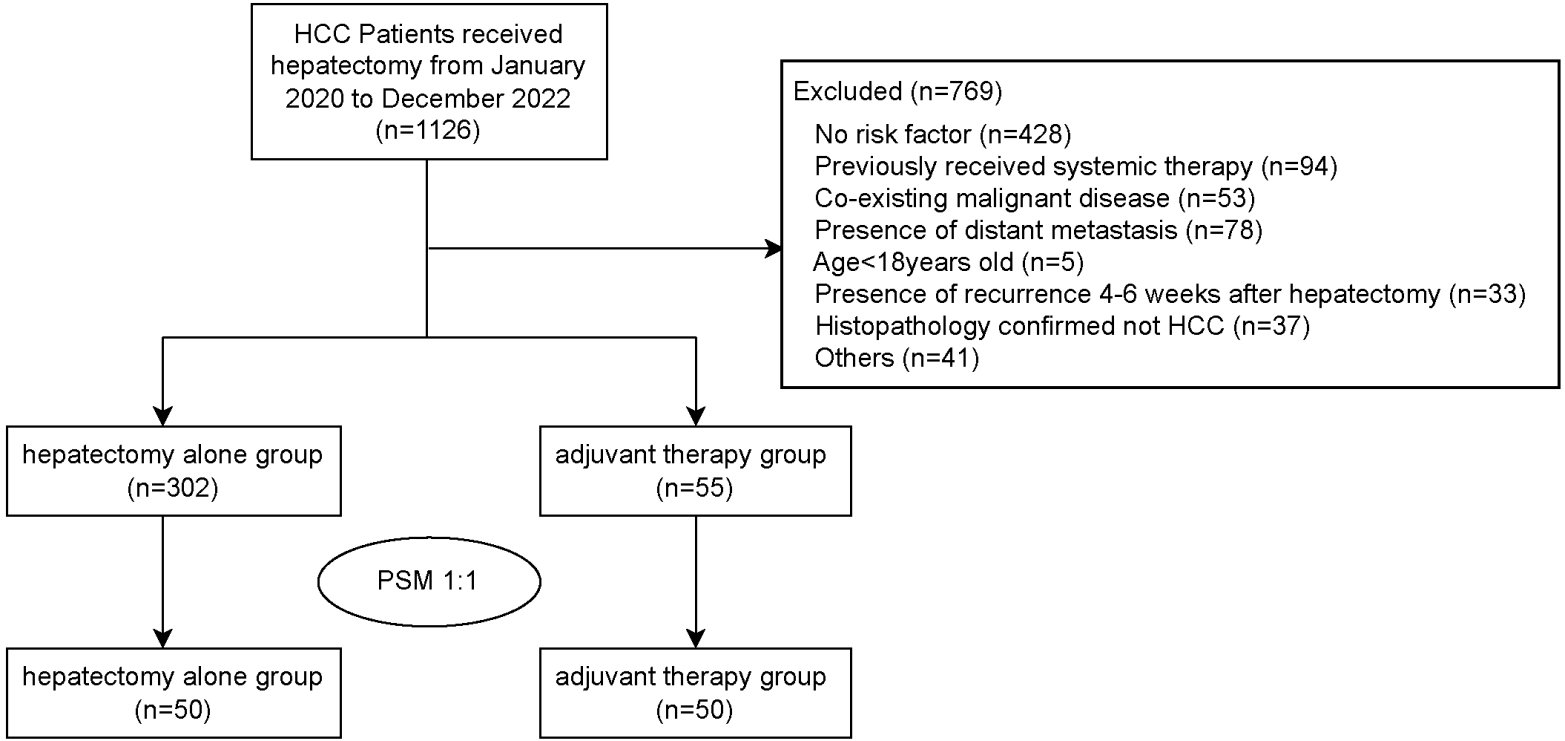
Flowchart of HCC patient enrollment.

Between January 2020 and December 2022, 1126 HCC patients underwent hepatectomy at the First Affiliated Hospital, Sun Yat-sen University. After applying inclusion and exclusion criteria, 769 patients were excluded and 357 patients were included (**Figure 1**). The main reason for exclusion included: absence of risk factors (n=428), prior systemic therapy (n=94), co-existing other malignancies (n=53), distant metastasis (n=78), age < 18 (n=5), early recurrence within 4–6 weeks post-hepatectomy (n=33), non-HCC histopathology (n=37), and other reasons (n=41).

Among the included patients, 302 underwent hepatectomy alone (HA group), and 55 received adjuvant TKIs combined with anti-PD-1 antibody (AT group). As shown in **Table 1**, significant differences in age, tumor size, adjuvant radiotherapy, and adjuvant interventional therapy (all *p* < 0.05) were observed when comparing the baseline characteristics of the two groups. To balance baseline characteristics, variables with a p-value less than 0.1 (age, PVTT, tumor size, adjuvant radiotherapy, and adjuvant interventional therapy) were used for 1:1 PSM, resulting in 50 matched pairs. After PSM, no statistical differences in variables were observed between the two groups, with all selected variables having an SMD less than 0.1. The majority of patients (>80%) in both groups were male, hepatitis B virus-infected, and Child-Pugh A after PSM.

**Table 1:** Baseline characteristics of all HCC patients (before and after PSM).

Variable	Before PSM				After PSM			
	HA group (n=302)	AT group (n=55)	P	SMD	HA group (n=50)	AT group (n=50)	P	SMD
Age (years)			0.0002	-0.6202			0.9622	0.0097
mean (SD)	55.7 (12.0)	49.4 (10.3)			50.0 (10.9)	50.1 (10.1)		
Gender (%)			0.2384				0.3173	
Female	36 (11.9)	3 (5.5)			7 (14.0)	3 (6.0)		
Male	266 (88.1)	52 (94.5)			43 (86.0)	47 (94.0)		
Lymph node invasion (%)			0.1328				1	
Negative	298 (98.7)	52 (94.5)			49 (98.0)	49 (98.0)		
Positive	4 (1.3)	3 (5.5)			1 (2.0)	1 (2.0)		
HVTT (%)			0.6385				1	
Negative	283 (93.7)	50 (90.9)			47 (94.0)	46 (92.0)		
Positive	19 (6.3)	5 (9.1)			3 (6.0)	4 (8.0)		
PVTT (%)			0.0688	0.26			0.8149	0.0852
Negative	240 (79.5)	37 (67.3)			39 (78.0)	37 (74.0)		
Positive	62 (20.5)	18 (32.7)			11 (22.0)	13 (26.0)		
Tumor size (%)			0.0185	-0.3391			1	0.0403
≤5cm	81 (26.8)	24 (43.6)			23 (46.0)	22 (44.0)		
>5cm	221 (73.2)	31 (56.4)			27 (54.0)	28 (56.0)		
Tumor number (%)			0.7283				0.1063	
1	207 (68.5)	37 (67.3)			39 (78.0)	34 (68.0)		
2	40 (13.2)	6 (10.9)			6 (12.0)	6 (12.0)		
3	3 (1.0)	0 (0.0)			2 (4.0)	0 (0.0)		
>3	52 (17.2)	12 (21.8)			3 (6.0)	10 (20.0)		
MVI (%)			0.6069				0.5365	
Negative	112 (37.1)	23 (41.8)			17 (34.0)	21 (42.0)		
Positive	190 (62.9)	32 (58.2)			33 (66.0)	29 (58.0)		
Satellite nodules (%)			1				0.7389	
Negative	268 (88.7)	49 (89.1)			46 (92.0)	44 (88.0)		
Positive	34 (11.3)	6 (10.9)			4 (8.0)	6 (12.0)		
Cirrhosis (%)			1				0.5433	
No	188 (62.3)	34 (61.8)			27 (54.0)	31 (62.0)		
Yes	114 (37.7)	21 (38.2)			23 (46.0)	19 (38.0)		
Child Pugh classification (%)			0.5535				1	
A	288 (95.4)	54 (98.2)			48 (96.0)	49 (98.0)		
B	14 (4.6)	1 (1.8)			2 (4.0)	1 (2.0)		
AFP>400 ng/mL (%)			0.6338				0.5201	
No	201 (66.6)	39 (70.9)			32 (64.0)	36 (72.0)		
Yes	101 (33.4)	16 (29.1)			18 (36.0)	14 (28.0)		
Adjuvant radiotherapy (%)			0.0293	0.2527			1	0
No	289 (95.7)	48 (87.3)			44 (88.0)	44 (88.0)		
Yes	13 (4.3)	7 (12.7)			6 (12.0)	6 (12.0)		
Adjuvant interventional therapy (%)			<0.0001	0.4785			1	0
No	284 (94.0)	40 (72.7)			40 (80.0)	40 (80.0)		
Yes	18 (6.0)	15 (27.3)			10 (20.0)	10 (20.0)		
HbsAg (%)			0.8626				1	
Negative	50 (16.6)	8 (14.5)			7 (14.0)	8 (16.0)		
Positive	252 (83.4)	47 (85.5)			43 (86.0)	42 (84.0)		
HCVAb (%)			0.8714				1	
Negative	298 (98.7)	55 (100.0)			49 (98.0)	50 (100.0)		
Positive	4 (1.3)	0 (0.0)			1 (2.0)	0 (0.0)		
BCLC classification (%)			0.2268				0.1499	
0	8 (2.6)	2 (3.6)			4 (8.0)	2 (4.0)		
A	158 (52.3)	21 (38.2)			30 (60.0)	21 (42.0)		
B	29 (9.6)	5 (9.1)			2 (4.0)	5 (10.0)		
C	107 (35.4)	27 (49.1)			14 (28.0)	22 (44.0)		

### Survival analysis

**Figure 2:**
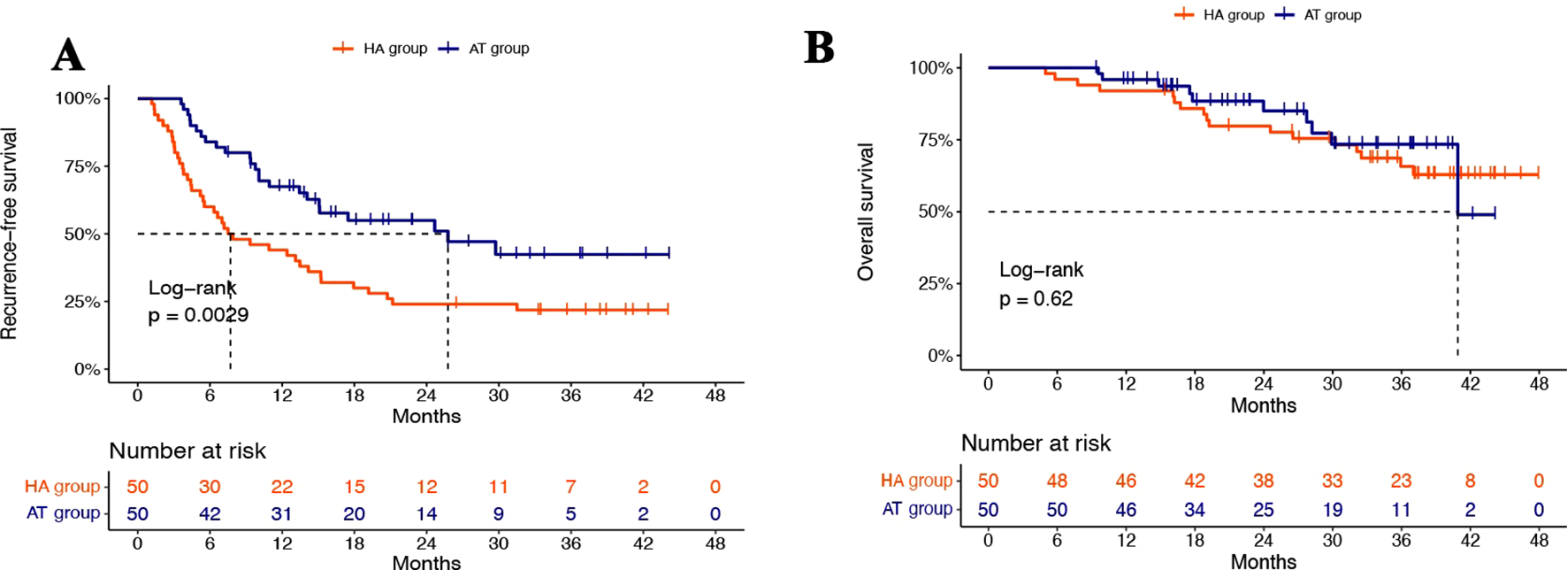
Survival curves for RFS **(A)** and OS **(B)** between AT group and HA group after PSM.

After PSM, the median follow-up was 30.2 months (IQR 18.47–37.48). Among the 100 matched patients, 63 (63%) experienced recurrence and 27 (27%) died. The median RFS was 25.77 months (95% CI: 15.07- not evaluated (NE)) in the AT group versus 7.7 months (95% CI: 5.43-15.2) in the HA group. The corresponding 1-year, 2-year, and 3-year RFS rates were 67.46%, 51.03%, and 42.40% in the AT group, compared to 42.0%, 24.0%, and 21.8% in the HA group, respectively. The RFS of patients in the AT group was significantly longer than that of patients in the HA group (*p* = 0.0029, **Figure 2A**). The median OS was 40.9 months (95% CI: 40.9- NE) in the AT group, while the HA group did not reach the median OS. The corresponding 1-year, 2-year, and 3-year OS rates were 95.91%, 85.03%, and 74.43% in the AT group, and 92.0%, 79.73%, and 68.61% in the HA group, respectively. There was no statistically significant difference in OS between the two groups (*p* = 0.62, **Figure 2B**).

### Univariable and multivariable analyses

As shown in **Table 2**, univariable analysis identified adjuvant TKIs combined with anti−PD−1 therapy (HR = 0.47, 95% CI = 0.28-0.78; *p* = 0.004), PVTT (HR = 2.34, 95% CI = 1.37-3.99; *p* = 0.002), BCLC stage (HR = 1.89, 95% CI = 1.14-3.11; *p* = 0.013) as significant predictors of RFS. In multivariable analysis, only adjuvant therapy remained an independent protective factor for RFS (HR = 0.38, 95% CI = 0.22-0.64; *p* < 0.001). For OS, univariate analysis showed PVTT (HR = 3.38, 95% CI = 1.59-7.21; *p* = 0.002) and BCLC stage (HR = 2.96, 95% CI = 1.37-6.41; *p* = 0.006) were significantly associated with survival prognosis. However, no independent predictors were identified in the multivariable analysis.

**Table 2:** Univariate and multivariate analysis of RFS and OS for HCC patients after PSM.

Variable	RFS				OS			
	Univariate Analysis		Multivariate Analysis		Univariate Analysis		Multivariate Analysis	
	HR (95% CI)	P	HR (95% CI)	P	HR (95% CI)	P	HR (95% CI)	P
Age								
>55 yr vs ≤55 yr	1.46 (0.87-2.44)	0.15			1.71 (0.79-3.68)	0.17		
Gender								
Male vs Female	1.50 (0.60-3.75)	0.38			3.60 (0.49-26.56)	0.21		
HVTT								
Positive vs Negative	1.37 (0.55-3.43)	0.50			1.56 (0.47-5.21)	0.47		
PVTT								
Positive vs Negative	2.34 (1.37-3.99)	0.002	2.08 (0.91-4.75)	0.083	3.38 (1.59-7.21)	0.002	2.3 (0.65-8.06)	0.19
Tumor size								
≥5cm vs ≤5cm	1.45 (0.87-2.41)	0.15			1.81 (0.79-4.13)	0.16		
Tumor number								
≥3 vs ≤3	1.42 (0.72-2.81)	0.31			1.73 (0.65-4.58)	0.27		
MVI								
Positive vs Negative	1.01 (0.61-1.68)	0.96			1.00 (0.46-2.19)	> 0.99		
Satellite nodules								
Positive vs Negative	1.48 (0.67-3.25)	0.33			2.43 (0.98-6.05)	0.055		
Cirrhosis								
Yes vs No	1.40 (0.85-2.30)	0.18			1.90 (0.89-4.05)	0.098		
Child Pugh classification								
B vs A	1.56 (0.38-6.45)	0.54			2.30 (0.54-9.73)	0.26		
AFP≥400 ng/mL								
Yes vs No	0.74 (0.42-1.30)	0.30			1.54 (0.71-3.31)	0.27		
Adjuvant radiotherapy								
Yes vs No	0.80 (0.37-1.77)	0.59			1.43 (0.49-4.15)	0.51		
Adjuvant interventional therapy								
Yes vs No	0.97 (0.53-1.79)	0.92			0.66 (0.25-1.76)	0.41		
HbsAg								
Positive vs Negative	1.12 (0.55-2.27)	0.75			0.63 (0.24-1.69)	0.36		
BCLC classification								
C vs 0/A/B	1.89 (1.14-3.11)	0.013	1.51 (0.70-3.30)	0.30	2.96 (1.37-6.41)	0.006	1.6 (0.45-5.77)	0.47
Adjuvant TKIs combined with anti–PD–1 antibody								
Yes vs No	0.47 (0.28-0.78)	0.004	0.38 (0.22-0.64)	< 0.001	0.82 (0.37-1.80)	0.62		

### Subgroup analysis

**Figure 3:**
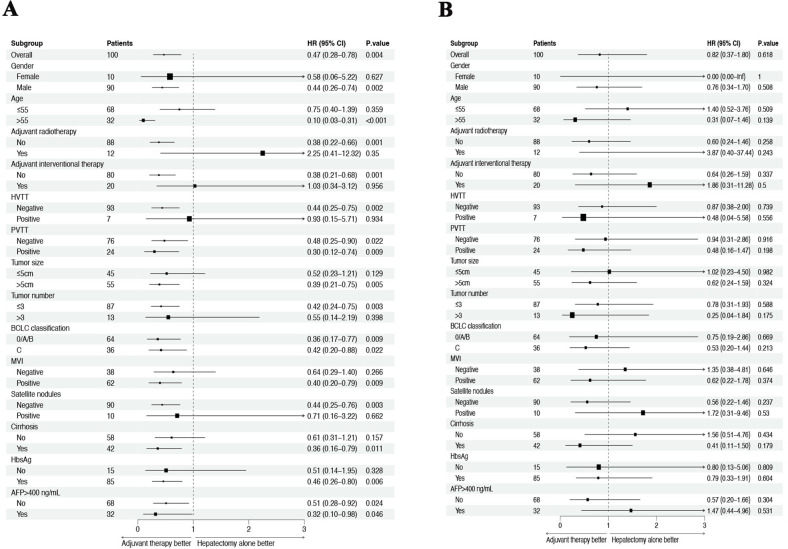
Forest plot for subgroup survival analysis for RFS **(A)** and OS **(B)**.

Subgroup analysis was performed to investigate the effect of adjuvant therapy on postoperative outcomes in HCC patients with high risk of recurrence. Results showed that adjuvant TKIs combined with anti-PD-1 antibody significantly improved RFS in male HCC patients (HR = 0.44, 95% CI = 0.26−0.74), those over 55 years old (HR = 0.10, 95% CI = 0.03−0.31), without adjuvant radiotherapy (HR = 0.38, 95% CI = 0.22−0.66), and without HVTT (HR = 0.44, 95% CI = 0.25−0. Other factors associated with longer RFS included: tumor size > 5cm (HR = 0.39, 95% CI = 0.21−0.75), tumor number ≤3 (HR = 0.42, 95% CI = 0.24−0.75), positive MVI (HR = 0.40, 95% CI = 0.20−0.79), negative satellite nodules (HR = 0.44, 95% CI = 0.25−0.76), presence of cirrhosis (HR = 0.36, 95% CI = 0.16−0.79), and positive HBsAg. (HR = 0.46, 95% CI = 0.26−0.80). The analysis also found that the AT group consistently achieved longer RFS, regardless of PVTT presence, BCLC stage less than C, or AFP greater than 400 ng/ml (**Figure 3A**). However, the AT group had similar OS compared to the HA group across all subgroups of HCC patients (**Figure 3B**).

## Discussion

Surgical resection remains the primary curative treatment for early-stage HCC. Despite curative resection, up to 70% of patients experience recurrence within 5 years, and the 5-year overall survival remains less than 50% (2, 3). High-risk factors, such as MVI, satellite nodules, PVTT, and HVTT are associated with poor prognosis, yet no standardized adjuvant therapy has been established for these patients. In this retrospective real-world study, we evaluated the efficacy and safety of adjuvant TKIs combined with anti−PD−1 antibody in high-risk HCC patients following hepatectomy. Our findings showed a significant improvement in RFS compared to surgery alone, although no OS benefit was observed. Multivariable analysis and subgroup analyses further supported the RFS benefit, highlighting the potential of this combination strategy to reduce early recurrence. Nonetheless, the absence of overall survival benefit necessitates cautious interpretation and highlights the need for extended follow-up.

The definition of “high-risk” recurrence profoundly influences study outcomes. Retrospective study has suggested RFS benefits of adjuvant sorafenib in high-risk patients (24). However, the STORM trial, which primarily enrolled early-stage liver cancer patients without high-risk recurrence factors, failed to demonstrate RFS or OS benefits (14). Similarly, HAIC and TACE have shown inconsistent outcomes depending on patient selection (25–28). These data emphasize the importance of precise risk stratification in adjuvant therapy. In line with this, our study adopted inclusion criteria consistent with a prior prospective observational study from our center (18, 29), which demonstrated RFS and OS benefits in patients with at least one high-risk feature. This study provided strong evidence that postoperative HCC patients with these high-risk recurrence factors can derive substantial benefit from adjuvant immunotherapy. Therefore, the selection criteria for the patient population in our current study were directly informed by the inclusion parameters from our center’s previous prospective observational study. This alignment ensures that our investigation is rooted in a robust framework for identifying patients most likely to benefit from adjuvant therapy, further advancing personalized treatment strategies for postoperative HCC.

Immune checkpoint inhibitors combined with anti-angiogenic agents represent a standard first-line therapy in advanced HCC (30–32), yet their role in the adjuvant setting remains under investigation. The IMbrave050 study aimed to evaluate the efficacy and safety of the atezolizumab plus bevacizumab (T+A) regimen compared to active surveillance in patients at high risk of recurrence following liver cancer surgery (19). The initial analysis demonstrated improved RFS with atezolizumab plus bevacizumab in high-risk postoperative HCC, leading to its inclusion in clinical guidelines. However, the final analysis showed attenuated RFS benefit and no OS trend, raising questions about its long-term value and prompting re-evaluation of its guideline recommendation (33). The lack of sustained benefit in IMbrave050 may be partly due to the limited duration of adjuvant therapy. While short-term treatment may reduce early recurrence, it is likely insufficient to prevent late recurrence driven by *de novo* tumorigenesis from chronic liver disease. Without long-term immunosurveillance, the impact on overall survival may be diminished.

Our study explored a different combination—PD-1 antibodies with TKIs—which may offer broader accessibility, lower cost, and favorable cost-effectiveness. The observed RFS benefit is consistent with previous studies on immune checkpoint inhibitors, either alone or in combination (16, 17, 34). However, OS benefits were not observed within our study’s follow-up period. Possible reasons include: firstly, follow-up limitations with a median follow-up of 30.2 months may be insufficient to capture OS benefits. After PSM, only 10 patients (20%) reached the endpoint in the adjuvant therapy group, compared to 17 patients (34%) in the non-adjuvant therapy group. A longer follow-up period is needed to ascertain the ultimate effect of adjuvant therapy. Secondly, despite propensity score matching (PSM), inherent biases in retrospective designs remain. Thirdly, patients who experienced tumor recurrence after adjuvant therapy might develop resistance to subsequent systemic treatment, leading to shortened PFS, which prevents the RFS benefit from translating into an OS benefit.

Currently, no clinical trials have directly compared ICIs alone versus ICIs combined with anti-angiogenic therapy in the adjuvant setting. Therefore, it remains unclear whether adding anti-angiogenic targeted therapy on the base of immune checkpoint inhibitors can further improve the prognosis of postoperative HCC patients. Our previous prospective observational study comes from the same center as this study, with consistent inclusion criteria and minimal differences in the enrolled population, making indirect comparison reasonably valid. In the previous study, the median RFS for patients treated with PD-1 antibody adjuvant therapy compared to observation was 17.76 vs. 5.73 months (P = 0.008), with a median RFS extension of approximately 12 months (18). In this study, the median RFS for PD-1 antibody combined with TKI adjuvant therapy compared to observation was 25.77 vs. 7.7 months (P = 0.0029), with a median RFS extension of about 18 months. This suggests that combining immune checkpoint inhibitors with anti-angiogenic targeted therapy as adjuvant therapy might be superior to using immune checkpoint inhibitors alone.

In this study, adverse events associated with PD-1 antibody and TKI combination therapy were manageable. Grade 3 side effects included elevated AST (8%) and ALT (10%), which resolved with appropriate management. No treatment discontinuations occurred due to toxicity, supporting the regimen’s tolerability.

This study has several limitations that should be acknowledged. First, as a retrospective observational study, it is inherently subject to methodological constraints. The decision to receive PD-1 antibody combined with TKI adjuvant therapy, as well as the choice of specific treatment drugs, was ultimately influenced by patients’ personal preferences. Factors such as differences in socioeconomic status and varying levels of acceptance of adjuvant therapy may have introduced selection bias. While propensity score matching (PSM) was applied to balance baseline variables and minimize the impact of covariates on outcomes, it is unlikely to eliminate all potential biases entirely. Secondly, the relatively small sample size (50 patients per group after PSM) and the short median follow-up period (30.2 months) limit the robustness of the study’s conclusions. These constraints may affect the generalizability of the findings and the ability to capture long-term outcomes. To address these limitations, future studies should focus on larger patient populations, longer follow-up periods, and ideally, prospective randomized controlled trials to validate the results of this study. These efforts will provide more definitive evidence and help guide clinical decision-making in adjuvant therapy for high-risk postoperative HCC patients.

## Conclusions

This study demonstrates that adjuvant tyrosine-kinase inhibitor plus PD-1 inhibitor was associated with longer recurrence-free survival in patients with high-risk hepatocellular carcinoma after curative resection. Although overall survival benefit was not observed within the follow-up period, the combination appears to be safe, accessible, and cost-effective. These findings support its potential clinical utility and underscore the need for prospective trials to confirm long-term efficacy and guide optimal patient selection.

## Data Availability

The raw data supporting the conclusions of this article will be made available by the authors, without undue reservation.
